# Effects of tocilizumab on renal function and oxidative stress in an experimental model of acute ischemic kidney injury^*^

**DOI:** 10.1590/1980-220X-REEUSP-2025-0220en

**Published:** 2025-12-19

**Authors:** Julia Matheus Tsuchiya, Juliana Veloso Gusmão Silva, Mykelly Gomes Alves, Eloiza de Oliveira Silva, Alessandra de Oliveira Maia, Maria de Fátima Fernandes Vattimo

**Affiliations:** 1 Universidade de São Paulo, Escola de Enfermagem, São Paulo, SP, Brazil.

**Keywords:** Acute Kidney Injury, Monoclonal Antibodies, Ischemia

## Abstract

**Introduction:**

Acute kidney injury (AKI) results from renal damage that triggers oxidative stress, inducing apoptosis, structural abnormalities in cells and their organelles, and even mitochondrial DNA instability. Tocilizumab is a monoclonal antibody inhibitor of interleukin-6, initially used to treat rheumatoid arthritis and later tested for COVID-19 treatment, which may have a protective effect on AKI.

**Objective:**

To evaluate the effect of tocilizumab on renal function and oxidative profile in rats with ischemic AKI.

**Methods:**

This is an experimental study using a quantitative approach with animals. The animals were randomized into four groups: SHAM (control); TCZ (tocilizumab); I/R (ischemia/renal reperfusion, clamping of both renal pedicles for 30 minutes); and TCZ + I/R. Tests were performed to assess renal function (inulin clearance and plasma creatinine), renal oxidation (urinary peroxides, malondialdehyde-derived oxidative substances), and endogenous antioxidant agents (thiols).

**Results:**

Regarding renal function, the treated group showed improvement in inulin clearance **(IR 0.24 ± 0.3 vs TCZ + IR 0.65 ± 0.05; p < 0.05)** and plasma creatinine levels **(IR 2.3 ± 0.6 vs TCZ + IR 0.8 ± 0.3; p < 0.05)**. Analysis of the oxidative profile revealed a reduction in peroxides, confirming an attenuation of the redox mechanism **(IR 15.8 ± 2.8 vs. TCZ + IR 3.7 ± 1.3; p < 0.05)**.

**Conclusion:**

Tocilizumab demonstrated renoprotective effects, improving renal function and reducing oxidative stress.

## INTRODUCTION

Acute kidney injury (AKI) is the abrupt reduction of renal function, defined by a decrease in the glomerular filtration rate (GFR) that results in a transient increase in renal waste products such as urea and creatinine, which may be accompanied by reduced urinary flow and fluid and electrolyte imbalances^(1)^.

Its classifications according to etiology include prerenal and renal, which may be associated with ischemic events such as sepsis, shock, infections, the use of radiographic contrast agents, and drug toxicity^(2)^. According to the Kidney Disease: Improving Global Outcomes (KDIGO) organization, one in five adults (21.6%) and one in three children (33.7%) worldwide develop AKI. Clinically, this syndrome is defined as an increase of ≥0.3 mg/dL or 1.5 times the baseline serum creatinine within 48 hours, or a reduction in urine output to less than 0.5 mL/kg for six consecutive hours^(3)^.

In ischemic conditions, where there are periods of prolonged reduction in renal blood flow leading to renal hypoperfusion—as in sepsis, cardiovascular surgeries, or allograft transplantation—the integrity and metabolism of renal cells are compromised, resulting in oxidative stress and, ultimately, vascular, glomerular, and tubular dysfunction, which together cause tissue injury in this organ^(3,4)^.

Ischemic injury is a major cause of decreased GFR, however, it does not always directly reflect the patient’s baseline condition, demonstrating the complexity of vascular and tubular processes involved in renal dysfunction^(4)^.

Some studies propose an experimental model that mimics ischemia-induced acute kidney injury (I-AKI), characterized by renal tissue hypoxia resulting from interruption of the local blood supply. In this model, ischemia is induced by clamping both renal pedicles, followed by reperfusion of renal blood flow^(4)^.

This renal injury technique causes mitochondrial damage, since these organelles depend on oxygen to perform oxidative phosphorylation, the primary mechanism of energy production. In the absence or suppression of oxygen, this process becomes anaerobic, leading to the accumulation of metabolites such as lactic acid. Excess lactic acid causes cellular acidosis and can damage mitochondrial membrane structure^(5–6)^. This cascade of cellular damage triggers inflammation as a consequence of the activation of the local pro-inflammatory immune response^(7)^.

Regarding defense cells and inflammatory agents, leukocytes, mast cells, and platelets stand out, releasing various types of lipid mediators (eicosanoids), proteins (cytokines and chemokines), and gaseous mediators (nitric oxide, carbon monoxide, reactive oxygen species)^(7)^. Inflammation has been considered the main mechanism of injury in ischemic lesions, such as I-AKI, and is directly associated with redox mechanisms.

In I-AKI, interleukin-6 (IL-6) plays a crucial role in both the initial inflammatory and recovery phases. During ischemia, a strong inflammatory response occurs, in which IL-6 is rapidly produced by endothelial cells, macrophages, and renal epithelial cells. The production of IL-6 is stimulated by local signals of cellular stress and hypoxia, promoting leukocyte activation and the release of other pro-inflammatory cytokines^(7–8)^.

The inflammatory mechanism in I-AKI involves increased infiltration of neutrophils and macrophages into the injured kidneys, exacerbating tissue damage. The IL-6 trans-signaling pathway, mediated by the soluble IL-6 receptor (sIL-6R), is particularly associated with amplification of the inflammatory process, worsening renal injury by enhancing the immune response, and triggering oxidative damage and apoptosis in renal cells^(8–9)^.

Based on this, the hypothesis of the present study is that tocilizumab (TCZ) can prevent AKI through its inhibitory action on IL-6, interfering with both antigen-specific immune responses and inflammatory processes, and acting as a key agent in the early stage of inflammation^(8)^.

In summary, TCZ is a monoclonal antibody that inhibits IL-6. It was initially used to treat rheumatoid arthritis and later tested for the treatment of acute respiratory infection caused by the SARS-CoV-2 coronavirus (COVID-19), due to its effects on inflammatory markers such as C-reactive protein, ferritin, and lactate dehydrogenase in patients receiving high-flow nasal cannula oxygen therapy or non-invasive ventilation^(8–9)^.

The present study follows a preclinical research model, whose results may inform clinical decision-making once incorporated into clinical and randomized trials, potentially leading to significant improvements in clinical practice^(10,11,12)^.

The nurse-researcher plays a vital role in translational medicine by developing and testing experimental models and innovations aimed at practical application and the improvement of clinical outcomes, such as novel pharmacological strategies to preserve renal function, as illustrated by the present project^(13)^.

Mastery of basic sciences is therefore essential for both the training of new researchers and advanced clinical practice, expanding the traditional model of “care” and reinforcing nursing’s role in the creation of innovative therapies^(13)^. Accordingly, this study aimed to evaluate the effect of tocilizumab on renal function and oxidative profile in rats with ischemic AKI.

## METHODS

### Ethical Aspects

The study was approved by the Ethics Committee on the Use of Animals of the School of Medicine, University of São Paulo (CEUA-FMUSP, as per its Portuguese acronym), under registration number CEUA: 2013/2023. Twenty adult male Wistar rats were used, provided by FMUSP. The study was conducted at the Experimental Animal Models Laboratory (LEMA, as per its Portuguese acronym) of the School of Nursing, University of São Paulo (EEUSP, as per its Portuguese acronym). The ethical aspects of the experimental protocol were based on the Brazilian Guidelines for the Care and Use of Animals for Scientific and Educational Purposes (DBCA, as per its acronym in Portuguese), in accordance with Law No. 11.794 of October 8, 2008, Decree No. 6.899 of July 15, 2009, and the standards established by the National Council for the Control of Animal Experimentation (CONCEA, as per its Portuguese acronym), as well as the ARRIVE 2.0 guidelines^(14)^.

### Experimental Design

Sample size was calculated using the G^*^Power software, which determined the number of animals required for the experiment. The adult male Wistar rats weighed between 250–300 g. The animals were randomized into four groups: SHAM Group (n = 5): Control animals subjected to laparotomy with simulated clamping of the renal pedicles; Tocilizumab Group (TCZ, n = 5): Animals that received tocilizumab (4 mg/kg, intraperitoneally, single dose) on the first day of the experimental protocol; Ischemia and reperfusion Group (I/R, n = 5): Animals subjected to bilateral clamping of the renal pedicles for 30 minutes, and TCZ + I/R Group (n = 5): Animals that received tocilizumab (4 mg/kg, intraperitoneally, single dose) on the first day of the protocol and underwent the ischemia procedure on the following day.

Pre-anesthesia was performed with morphine (2%, 2 mg/kg, intramuscularly, single dose). After 10 minutes, anesthesia was induced with isoflurane (5% for induction and 3% for maintenance in 2 L of O_2_). Laparotomy was performed for bilateral clamping of the renal pedicles using atraumatic vascular clamps. All animals were sutured with 3.0 mononylon thread, monitored during anesthetic recovery, and received postoperative analgesia with tramadol (15 mg/kg intramuscularly, three times daily for three days). On the third day, they were placed in metabolic cages for the assessment of renal function and oxidative profile.

On the fourth day, the animals were anesthetized as described above (morphine followed by isoflurane) and positioned on a heated surgical table. Tracheostomy was performed to maintain airway patency. The left jugular vein was dissected for inulin infusion over two hours, with blood samples collected every 60 minutes, and the carotid artery was cannulated for measurement of mean arterial pressure. Laparotomy was also performed for bladder drainage and urine collection every 30 minutes, followed by dissection or puncture of the abdominal aorta. The left kidney was removed, stored at -80° C, and preserved for later analysis of non-protein thiol levels.

### Assessment of Oxidative Stress

The evaluations were performed using plasma, urine, and renal tissue samples collected and stored after euthanasia, according to the following analyses:

Urinary peroxides: were measured using the ferrous oxidation-xylenol orange (FOX) assay, which directly quantifies peroxides based on the oxidation of ferrous ions in the presence of xylenol orange, enabling the determination of urinary peroxide levels^(15–16)^.

Lipid peroxidation: was assessed using the thiobarbituric acid reactive substances (TBARS) assay, which quantifies malondialdehyde (MDA), a major product of this oxidative cascade. MDA reacts with thiobarbituric acid, forming a colored complex that can be spectrophotometrically measured^(17–18)^.

Urinary nitrate synthesis: was evaluated through quantification of nitrite (NO_2_-), a stable metabolite of nitric oxide (NO), using the Griess reaction. This colorimetric reaction is based on the interaction of nitrites with sulfanilic acid, followed by coupling with α-naphthylamine hydrochloride in an acidic medium (pH 2.5–5.0), forming a pink-colored compound (α-naphthylamine-p-azobenzene-p-sulfonic acid). Approximately 150 µL of urine from each experimental group was mixed with 150 µL of Griess reagent and incubated for 15 minutes. Absorbance was measured at 545 nm using an ELISA plate reader, and values were compared with a sodium nitrite (NaNO_2_) standard curve ranging from 0.1 to 1.0 M^(18)^.

Thiol quantification (antioxidant response): was used as an indicator of the antioxidant response, based on the principle that a higher degree of oxidative stress results in increased oxidation of thiols and, consequently, lower thiol concentrations in renal tissue^(18)^.

### Statistical Analysis

Data analysis was performed using GraphPad Prism 8.0 software. One-way ANOVA was applied, with a significance level set at p < 0.05, considering that at least one group differed from the others. Tukey’s post hoc multiple-comparison test was used to assess pairwise differences between group means, based on the minimum significant difference and the assumption of homogeneity of variances across groups.

## RESULTS

### Renal Function Parameters

**Figure 1:**
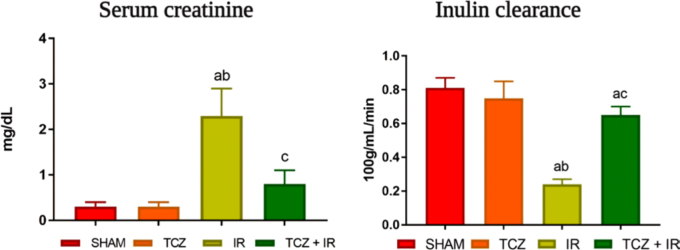
Renal function in the different experimental groups – São Paulo, SP, Brazil, 2024.

The renal function parameters are presented in Figure 1. Data from the SHAM group were used as the control and considered the baseline reference for normal renal function. Similarly, the TCZ group showed renal function results (inulin clearance) comparable to those of the SHAM group, confirming that tocilizumab alone did not affect renal function.

The I/R group confirmed the I-AKI, as evidenced by the increase in plasma creatinine and the decrease in inulin clearance (p < 0.05) (Table 1, Figure 1). In contrast, the TCZ + I/R group demonstrated a significant reduction in plasma creatinine (p < 0.05) and a marked increase in inulin clearance compared with the I/R group (Figure 1, Table 1).

**Table 1:** Renal function of the different experimental groups – São Paulo, SP, Brazil, 2024.

Group	n	Serum creatinine (mg/dL)	n	Inulin clearance (mL/min/100 g)
SHAM	5	0.3 ± 0.1 [0.2–0.4]	5	0.81 ± 0.06 [0.8–0.9]
TCZ	5	0.3 ± 0.1 [0.2–0.4]	5	0.75 ± 0.1 [0.7–0.8]
I/R	5	2.3^a b^ ± 0.6 [1.7–2.8]	5	0.24^a b^ ± 0.03 [0.2–0.3]
TCZ + I/R	5	0.8^c^ ± 0.3 [0.5–1.1]	5	0.65^c^ ± 0.05 [0.6–0.7]

Source: Author’s data, 2024.

Legend: ^a^p < 0.05 versus SHAM.

^b^versus TCZ.

^c^versus I/R. Values are expressed as mean ± standard deviation (SD), followed by the 95% confidence interval (CI). Statistical analysis revealed significant differences: Serum creatinine: increased in the I/R group (2.3 mg/dL; IC95% [1.7–2.8]) compared with SHAM and TCZ; reduced in the TCZ + I/R (0.8 mg/dL; IC95% [0.5–1.1]) compared to the I/R group. Inulin clearance: decreased in the I/R group (0.24 mL/min/100g; IC95% [0.2–0.3]) compared with SHAM and TCZ; increased in the TCZ + I/R (0.65 mL/min/100g; IC95% [0.6–0.7]) compared to the I/R group.

### Oxidative Profile

**Figure 2:**
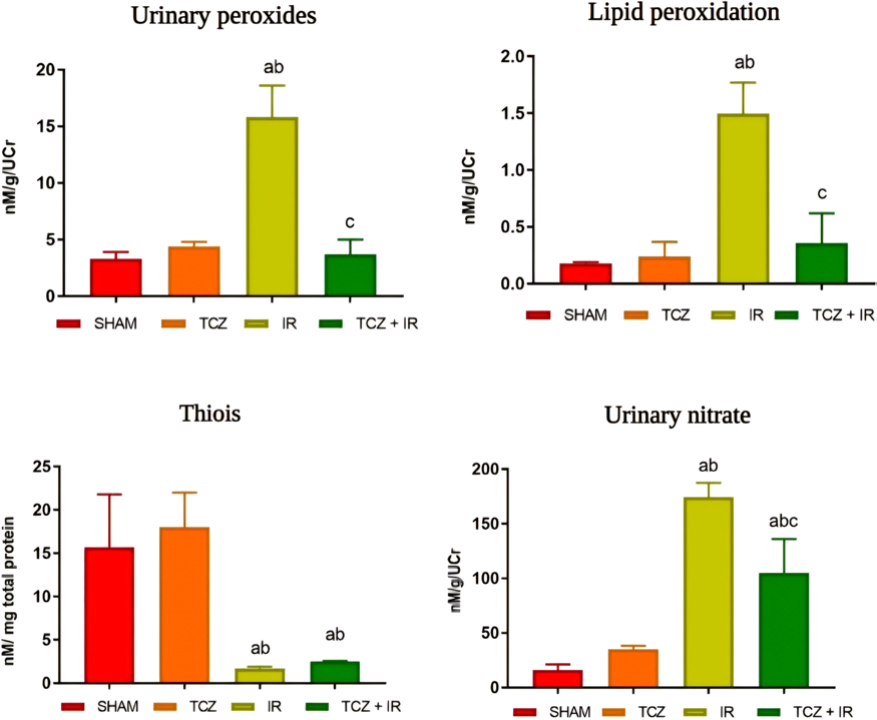
Oxidative stress profile – São Paulo, SP, Brazil, 2024.

The oxidative profile parameters are shown in Figure 2. As demonstrated, the SHAM group was used as the reference for normal values. The TCZ group showed data similar to those of the SHAM group.

For urinary peroxides, lipid peroxidation, and urinary nitrate, the I/R group showed higher mean values than the control groups (SHAM and TCZ). The TCZ + I/R group showed a marked reduction in oxidative metabolites compared with the I/R group.

This finding demonstrates a similar oxidative response between the controls and the TCZ + I/R. Regarding the antioxidant response, both I/R and TCZ + I/R groups presented significantly lower (p < 0.05) values than the controls, with no relevant difference between them (Figure 2, Table 2).

**Table 2:** Oxidative profile of the experimental animals – São Paulo, SP, Brazil, 2024.

Group	n	Urinary peroxides – FOX 2 (nM/g of urinary creatinine)	n	Lipid peroxidation – TBARS (nM/g of urinary creatinine)	n	Urinary nitrate (nmol/g urinary creatinine)	n	Thiols (nmol/mg total protein)
SHAM	5	3.3 ± 0.6	5	0.18 ± 0.01	5	16.0 ± 5.2	5	15.7 ± 6.1
TCZ	5	4.4 ± 0.4	7	0.24 ± 0.13	5	34.9 ± 3.4	5	18.0 ± 4.0
I/R	5	15.8^a b^ ± 2.8	6	1.50^a bd^ ± 0.27	5	174.5^a bd^ ± 13.0	5	1.7^a b^ ± 0.2
TCZ + I/R	5	3.7^c^ ± 1.3	5	0.36^c^ ± 0.26	5	105.3^a b c^ ± 30.8	5	2.5^a b^ ± 0.1

Source: São Paulo, SP, Brazil 2024. Author’s data.

Legend: ^a^p < 0.05 versus SHAM

^b^versus TCZ;

^c^versus I/R. Original data followed by mean and standard deviation. The table shows significant expression in the analysis of data from the I/R and TCZ + I/R groups in: urinary peroxides (15.8 nM/g of urinary creatinine and 3.7 nM/g of urinary creatinine, respectively); lipid peroxidation (1.50 nM/g of urinary creatinine and 0.36 nM/g of urinary creatinine, respectively); and urinary nitrate (174.5 nM/g of urinary creatinine and 105.3 nM/g of urinary creatinine, respectively).

## DISCUSSION

It is important to analyze the scope of this study, which evaluated renal function parameters (inulin clearance) and oxidative stress markers in animals with acute ischemic kidney injury (I-AKI). The results demonstrated that treatment with TCZ, a humanized monoclonal antibody against the interleukin-6 (IL-6) receptor used in inflammatory conditions, significantly attenuated renal damage caused by the ischemia–reperfusion syndrome induced by renal pedicle clamping. This effect was evidenced by an increase in inulin clearance and reduced oxidative stress.

In this study, the animals were subjected to tissue hypoperfusion—a process known as ischemia—which mimics physiological conditions observed in sepsis, acute coronary syndromes, and organ transplantation, followed by restoration of blood flow (reperfusion). This procedure induces tissue injury, triggering the activation of inflammatory cytokines and oxidative stress^(19)^.

Erdem et al. demonstrated that I-AKI damage is mediated by oxidative molecules, such as monoaldehyde, and by inflammatory mediators, including NF-κB, TNF-α, IL-6, and IL-1β. IL-6 plays a dual role in renal cell injury and repair and is particularly involved in immune, metabolic, ischemic, and toxic alterations of the kidneys^(19)^.

The present study confirmed that I-AKI resulted from renal pedicle clamping. In this context of hypoperfusion-induced injury, pre-treatment with TCZ attenuated the ischemia-related functional impairment, as demonstrated by the increase in the gold-standard marker of renal function used here—inulin clearance^(20)^.

The role of IL-6 in renal function and the pharmacological potential of tocilizumab have been described in cases of acute glomerulonephritis and renal transplantation, in which IL-6 acts as a pro-inflammatory mediator^(21–22)^. IL-6 plays a central role by regulating inflammatory processes and mediating the activation and maturation of T cells, B cells, and plasma cells—mechanisms closely related to transplant rejection. Previous studies, such as that by Jordan et al. (2017), demonstrated that IL-6 inhibition significantly reduces graft loss, reinforcing the therapeutic potential of TCZ in renal injury contexts^(23)^.

In an experimental study from Turkey using rats subjected to one hour of ischemia followed by six hours of reperfusion, the animals were divided into three groups—ischemic and ischemic + TCZ pre-treatment. The treated group showed reduced urea and creatinine levels, consistent with our model, which used inulin clearance as the functional marker^(23)^.

According to Fukuda (2021), in a clinical case report involving patients with renal impairment treated with tocilizumab, renal function was preserved and the need for renal replacement therapy was avoided, confirming the drug’s renoprotective effect. However, there is still no consensus on the prophylactic use of TCZ in renal diseases^(24,25,26,27,28)^.Clinical trials using TCZ have reported promising outcomes in renal transplantation, where antibody-mediated rejection is a major post-transplant complication^(28–29)^.

In this regard, the results presented here are consistent with both experimental and clinical evidence showing that tocilizumab not only modulates inflammation through the IL-6 pathway but also significantly reduces oxidative stress, protecting against cellular damage and vascular dysfunction. The reduction in oxidative stress likely contributed to decreased inflammation^(28–29)^.

Analysis of oxidative stress markers revealed normal values in the SHAM and TCZ groups, while renal dysfunction was evident in the I/R group. The TCZ + I/R group exhibited a marked reduction in lipid peroxidation and urinary peroxides, suggesting that TCZ effectively reduced reactive oxygen species (ROS) and may have improved mitochondrial response under ischemic conditions^(28–29)^. Additionally, regarding urinary nitrate, the TCZ + I/R group showed significant differences compared with both the control and I/R groups, confirming tocilizumab’s ability to decrease reactive nitrogen species^(28–29)^.

Conversely, some authors studying microarray expression have shown that TCZ, in patients treated for juvenile idiopathic arthritis, may decrease mitochondrial membrane potential and ATP production, leading to increased intracellular ROS levels. There is growing evidence that mitochondrial ROS and defective antioxidant responses play key roles in the pathogenesis of various inflammatory and autoimmune diseases^(28,29,30)^.

Therefore, the present study supports the conclusion that tocilizumab exerts antioxidant and renoprotective effects, attenuating kidney injury triggered by ischemia–reperfusion syndrome. These findings are consistent with the existing literature and suggest that TCZ could represent a therapeutic strategy in conditions where ischemia is already established, such as sepsis, or during renal transplantation surgeries, to mitigate ischemic injury to the graft and optimize postoperative functional recovery^(29–30)^.

In the context of experimental research, it is important to highlight that this work generated basic-science evidence that serves as a crucial foundation for nursing practice. Integrating fundamental sciences such as physiology, molecular biology, and animal experimentation enhances nurses’ ability to interpret pathophysiological mechanisms and to design safer, evidence-based interventions at the bedside^(29–30)^.

Thus, participation in translational medicine is integral to nursing practice and directly linked to human physiology as derived from basic research. This integration strengthens evidence-based decision-making and raises the standard of care in managing acute kidney injury^(29–30)^.

### Limitations

As this was an animal experimental model, its immediate applicability to clinical practice is limited. However, the findings provide a valuable foundation for future human studies, such as randomized clinical trials, which may ultimately influence public health policies and support the official inclusion of tocilizumab and similar agents in the national pharmacological arsenal for conditions in which inflammation is a primary pathogenic mechanism.

### Future Implications

Future perspectives for this research include expanding on current findings and thoroughly elucidating the mechanisms of action of tocilizumab in ischemic acute kidney injury (I-AKI). Moreover, long-term evaluations are warranted to investigate TCZ’s impact on renal recovery and on the prevention of chronic renal damage following I-AKI, both in animal models and subsequently in clinical studies.

## CONCLUSION

Tocilizumab showed promise in protecting the kidney in a preclinical model of ischemia and reperfusion, as evidenced by improvements in renal function, as measured by serum creatinine and inulin clearance. Regarding the oxidative profile, this drug showed an antioxidant effect, reducing urinary peroxides, lipid peroxidation, and urinary nitrate in the I-AKI model described in the study.

## Data Availability

The entire dataset supporting the results of this study was published in the article itself.
