# Supplementary material for: Effects of tocilizumab on renal function and oxidative stress in an experimental model of acute ischemic kidney injury
Source: Rev Esc Enferm USP. 2025 Dec 19;59:e20250220. doi: 10.1590/1980-220X-REEUSP-2025-0220en (PMC12815415; doi:10.1590/1980-220X-REEUSP-2025-0220en)
Supplement: Translation

# Translation: Efeitos do Tocilizumabe sobre a função renal e o estresse oxidativo em modelo experimental de injúria renal aguda isquêmica^*^

**DOI:** 10.1590/1980-220X-REEUSP-2025-0220pt

**Authors:** Julia Matheus Tsuchiya, Juliana Veloso Gusmão Silva, Mykelly Gomes Alves, Eloiza de Oliveira Silva, Alessandra de Oliveira Maia, Maria de Fátima Fernandes Vattimo

**Affiliations:** 1 Universidade de São Paulo, Escola de Enfermagem, São Paulo, SP, Brasil.

**Keywords:** Injúria Renal Aguda, Anticorpos Monoclonais, Isquemia

## Abstract

**Introdução:**

A Injúria Renal Aguda (IRA) deriva de insultos renais que desencadeiam o estresse oxidativo, apoptose, anomalias estruturais nas células e suas organelas e até mesmo instabilidade no DNA mitocondrial. O tocilizumabe é um anticorpo monoclonal inibidor da interleucina-6, inicialmente utilizado para tratamento de artrite reumatoide e, posteriormente, usado para o tratamento de COVID-19 que pode ter efeito de proteção na IRA.

**Objetivo:**

Avaliar o efeito do tocilizumabe sobre a função e o perfil oxidativo renal de ratos com IRA’ isquêmica.

**Métodos:**

Trata-se de um estudo experimental com animais de abordagem quantitativa. Os animais foram randomizados em 4 grupos: SHAM (controle); TCZ (tocilizumabe); I/R (isquemia/reperfusão renal por clampeamento dos pedículos renais por 30 minutos) e TCZ + I/R. Foram feitas provas de função renal (*clearance* de inulina, creatinina plasmática) e oxidação renal (peróxidos urinários, substâncias oxidativas derivadas do ácido malondealdeído) e agentes antioxidantes endógenos (tióis).

**Resultados:**

Quanto à função renal, o grupo tratado apresentou elevação do *clearance* de inulina **(IR 0,24 ± 0,3 vs TCZ + IR 0,65 ± 0,05; p < 0,05)** e da creatinina plasmática **(IR 2,3 ± 0,6 vs TCZ + IR 0,8 ± 0,3; p < 0,05)**. A análise do perfil oxidativo demonstrou redução dos peróxidos, confirmando uma atenuação do mecanismo redox **(IR 15,8 ± 2,8 vs TCZ + IR 3,7 ± 1,3; p < 0,05)**.

**Conclusão:**

O tocilizumabe apresentou um efeito renoprotetor, com melhora da função renal e redução do estresse oxidativo.

## INTRODUÇÃO

A Injúria Renal Aguda (IRA) é a redução abrupta da função renal, definida pela diminuição da Taxa de Filtração Glomerular (TFG), resultando em aumento transitório das escórias renais, como ureia e creatinina, podendo ser acompanhada de redução do fluxo urinário e de desequilíbrios hidroeletrolíticos^(1)^.

Dentre as suas classificações, segundo a etiologia, estão descritas a pré-renal e renal, que podem estar associadas a eventos de isquemia, como sepse, choque, infecções, uso de contrastes radiológicos e toxicidade por medicamentos^(2)^. Segundo a organização *Kidney Disease: improving global outcomes* (KDIGO), 1 em cada 5 adultos (21,6%) e 1 em cada 3 crianças (33,7%) desenvolvem IRA no mundo. Clinicamente, essa síndrome é definida como aumento de 0,3 mg/dL ou 1,5 vezes na creatinina sérica basal em 48h ou redução do fluxo urinário para menos de0,5 mL/Kg durante 6 h^(3)^.

Em cenários de isquemia onde há a ocorrência de períodos de redução prolongada do fluxo sanguíneo causando a hipoperfusão renal, como em casos de sepse, cirurgias cardiovasculares ou transplantes de aloenxertos, ocorre o comprometimento da integridade e do metabolismo celular renal e estresse oxidativo e, em última instância, as disfunções vasculares, glomerulares e tubulares, o que gera um insulto nesse órgão^(3,4)^.

A lesão por isquemia é causa da redução da TFG, porém, esta nem sempre se relaciona diretamente com a condição basal do paciente, o que mostra a complexidade dos processos vasculares e tubulares na disfunção renal^(4)^.

Alguns estudos propõem um procedimento de mimetização da Injúria Renal Aguda gerada por Isquemia (IRAi) que consiste em hipóxia nos tecidos renais, causada pela interrupção da irrigação sanguínea local. Nesse caso, a isquemia é induzida por meio do clampeamento dos pedículos renais, seguido de reperfusão do fluxo sanguíneo no tecido renal^(4)^.

Essa técnica de insulto renal causa danos mitocondriais, pois essas organelas dependem de oxigênio para realizar a produção dos processos de fosforilação oxidativa, o principal mecanismo de produção de energia. Na ausência ou supressão do oxigênio, esse processo ocorre de forma anaeróbica e resulta na formação de produtos, como o ácido lático. Esse ácido, quando acumulado, gera acidose celular e pode ocasionar danos na estrutura da membrana mitocondrial^(5–6)^. Esse conjunto de danos celulares resulta na inflamação, consequência da ativação do sistema imune pró-inflamatório próximo ao local^(7)^.

No que se refere às células de defesa e aos agentes inflamatórios, destacam-se os leucócitos, mastócitos e plaquetas, que liberam vários tipos de mediadores lipídicos (eicosanoides), proteínas (citocinas e quimiocinas) e mediadores gasosos (óxido nítrico, monóxido de carbono, espécies reativas de oxigênio)^(7)^. A inflamação tem sido considerada o principal mecanismo de lesão nas lesões isquêmicas como a IRAi e está diretamente envolvida com o mecanismo redox.

Na IRAi, a interleucina-6 (IL-6) desempenha um papel importante tanto na fase inicial de inflamação quanto na fase de recuperação. Durante a isquemia, ocorre uma resposta inflamatória significativa na qual a IL-6 é rapidamente produzida pelas células endoteliais, macrófagos e células epiteliais renais. A produção de IL-6 é estimulada por sinais locais de estresse celular e hipóxia, promovendo a ativação de leucócitos e a liberação de outras citocinas pró-inflamatórias^(7–8)^.

O mecanismo inflamatório na IRAi envolve o aumento da infiltração de neutrófilos e macrófagos nos rins lesionados, o que agrava o dano tecidual. A via de trans-sinalização da IL-6, mediada pelo receptor solúvel de IL-6 (sIL-6R), está particularmente associada à intensificação do processo inflamatório, agravando a lesão renal ao amplificar a resposta imune e causar dano oxidativo e apoptose nas células renais^(8–9)^.

Nesse sentido, a hipótese deste estudo é que o tocilizumabe (TCZ) pode prevenir a IRA pela ação inibitória sobre a IL-6, interferindo tanto nas respostas imunológicas específicas a antí-genos quanto nos processos inflamatórios, posicionando-se como um dos principais agentes da etapa inicial da inflamação^(8)^.

Sumariamente, o TCZ é um anticorpo monoclonal inibidor da IL-6. Ele foi inicialmente utilizado para tratamento de artrite reumatoide e, posteriormente, testado para tratamento da infecção respiratória aguda causada pelo coronavírus SARSCoV-2 (COVID-19), por seus efeitos no perfil de marcadores inflamatórios, como proteína C-reativa, ferritina e desidrogenase lática em pacientes com cânula nasal de alto fluxo e ventilação não invasiva^(8–9)^.

O estudo ora apresentado segue um modelo de pesquisa pré-clínica cujos resultados podem subsidiar decisões assistenciais após sua incorporação em estudos clínicos e ensaios randomizados, podendo resultar em relevantes melhorias na prática clínica^(10,11,12)^.

O enfermeiro pesquisador tem um papel vital na medicina translacional, ao desenvolver e testar modelos experimentais e inovações que têm como objetivo final a aplicação prática e a melhoria dos resultados clínicos, como no caso de novos fármacos, para preservar a função renal à semelhança do projeto ora apresentado^(13)^.

O domínio das ciências básicas, portanto, é essencial tanto à formação de novos pesquisadores quanto à prática avançada, ampliando o modelo tradicional do “cuidar” e legitimando a participação da enfermagem na criação de terapias inovadoras^(13)^. Sendo assim, a vigente pesquisa teve o objetivo de avaliar o efeito do tocilizumabe sobre a função e o perfil oxidativo renal de ratos com IRA isquêmica.

## MÉTODOS

### Aspectos Éticos

O estudo foi aprovado pelo Comitê de Ética para Uso de Animais da Faculdade de Medicina da Universidade de São Paulo (CEUA-FMUSP), sob o registro CEUA: 2013/2023. Foram utilizados 20 ratos Wistar, machos e adultos, fornecidos pela Faculdade de Medicina da USP. O estudo foi desenvolvido no Laboratório Experimental de Modelos Animais (LEMA) da Escola de Enfermagem da Universidade de São Paulo (EEUSP). O aspecto ético do protocolo experimental baseou-se na Diretriz Brasileira para o Cuidado e a Utilização de Animais para fins Científicos e Didáticos – DBCA. Seguindo o acordo com os preceitos da Lei nº 11.794, de 8 de outubro de 2008, do Decreto nº 6.899, de 15 de julho de 2009 e com normas editadas pelo Conselho Nacional de Controle de Experimentação em Animais (CONCEA) e do guia ARRIVE 2.0^(14)^.

### Delineamento Experimental

O cálculo amostral utilizado no estudo foi realizado por meio do software G*power*,* que recomenda o número de animais necessários ao experimento. Os ratos Wistar, machos e adultos, pesavam entre 250 e 300 g. Os animais foram randomizados em quatro grupos: Grupo SHAM (n = 5): animais controle submetidos à laparotomia para simulação do clampeamento dos pedículos renais; Tocilizumabe (TCZ, n = 5): animais que receberam Tocilizumabe (4 mg/kg, i.p., dose única), no 1° dia do protocolo experimental; Isquemia e reperfusão (I/R, n = 5): animais submetidos à laparotomia para o clampeamento bilateral dos pedículos renais por 30 minutos; e TCZ + I/R (n = 5): animais que receberam o Tocilizumabe (4 mg/kg, i.p., dose única) no 1° dia de protocolo e, no 2º dia, foram submetidos ao procedimento de I/R.

Os animais receberam pré-anestésico com morfina (2%, 2 mg/kg, uma vez, intramuscular) e, após 10 min, foram anestesiados com isoflurano (5% dose de indução e 3% dose de manutenção à 2 L de O_2_) e submetidos a laparotomia para clampeamento bilateral dos pedículos renais com *clamps* vasculares não traumáticos. Todos os animais foram suturados com fio *mononylon* 3.0, acompanhados durante a recuperação anestésica e receberam protocolo de analgesia no pós-operatório com tramadol (15 mg/Kg intramuscular, 3 vezes ao dia, 3 dias). No 3º dia, foram colocados em gaiola metabólica para análise da função renal e perfil oxidativo.

No 4º dia, os animais foram anestesiados conforme descrito acima (morfina seguida de isoflurano) e colocados em mesa cirúrgica aquecida. Realizou-se uma traqueostomia para liberação das vias aéreas superiores. Em seguida, procedeuse à dissecção da veia jugular esquerda para infusão de inulina durante 2 horas e coleta de amostras de sangue a cada 60 minutos, além da canulação da carótida direta para mensuração da pressão arterial média. Foi realizada também uma laparotomia para drenagem da bexiga e coleta de amostras de urina a cada 30 minutos, seguida da dissecção ou punção da aorta abdominal. O rim esquerdo foi removido, acondicionado e armazenado em refrigerador a -80º C para estudos posteriores de mensuração de tióis não proteicos.

### Avaliação do Estresse Oxidativo

As avaliações foram feitas nas amostras de plasma, urina e tecido renal extraídas e armazenadas após a eutanásia, obedecendo as análises a seguir:

Peróxidos urinários: foram avaliados pelo ensaio de oxidação ferrosa de laranja de xilenol (FOX), que faz mensuração direta de peróxidos e consiste na utilização de ferro-xilenol laranja para determinação dos níveis de peróxidos urinários^(15–16)^.

Peroxidação lipídica: foi analisada pelo ensaio de substâncias reativas ao ácido tiobarbitúrico (TBARS), o qual mensura o malondealdeído (MDA) por ser um dos principais produtos dessa cascata. Um método para detecção desse produto é a dosagem do ácido tiobarbitúrico ao qual ele reage^(17–18)^.

Síntese de nitrato urinário: foi avaliada por meio da quantificação de nitrito (NO2^-^), metabólito estável do NO, pelo método de Griess. A reação é colorimétrica e baseia-se na reação dos nitritos com ácido sulfanílico e copulação com cloridrato de alfa-naftilamina em meio ácido (pH entre 2,5 e 5,0) formando o ácido alfa-naftilamino-p-azobenzeno-p-sulfônico de coloração rósea. Cerca de 150 µl da amostra urinária dos diversos grupos foram adicionados a 150 µl do reagente que permaneceu em repouso por 15 min. A leitura foi realizada em absorbância de 545 nm, em leitor de ELISA. A absorbância das amostras foi comparada a uma curva padrão de nitrito de sódio 15 (NaNO^2^) na concentração de 0,1 a 1,0 M^(18)^.

Quantificação de tióis (antioxidantes): foi utilizada como indicador de resposta antioxidante, considerando o seguinte princípio: quanto maior o grau de estresse oxidativo, maiores foram os níveis de tióis oxidados e menores foram as concentrações de tióis no tecido renal^(18)^.

### Análise Estatística

A análise dos dados foi realizada no software GraphPad Prism versão 8.0, por meio do método ANOVA. Adotou-se um nível de significância de p < 0,05, considerando-se que o efeito de pelo menos um dos grupos diferisse dos outros. E os testes múltiplos de comparação 2 a 2 feitos pelo teste de Tukey, comparando os possíveis pares de médias baseando-se na diferença mínima significativa na homogeneidade das variâncias que diferem ou não entre si nos grupos.

## RESULTADO

### Parâmetros de Função Renal

Os parâmetros de função renal estão apresentados na Figura 1. Os dados do SHAM foram utilizados como grupo controle, considerados referência de normalidade. Nessa mesma lógica, o grupo TCZ apresentou dados de função renal (*clearance* de inulina) semelhantes àqueles demonstrados pelo grupo SHAM, confirmando que o tocilizumabe, isoladamente, não afetou a função renal.

**Figura 1:** Função renal dos diferentes grupos experimentais – São Paulo, SP, Brasil, 2024.

O grupo I/R confirmou a IRAi considerando a elevação da creatinina e a diminuição do *clearance* de inulina (p < 0,05) (Tabela 1 e Figura 1). O grupo TCZ + I/R demonstrou redução da creatinina plasmática (p < 0,05) e elevação relevante no *clearance* de inulina na comparação com o grupo I/R. (Figura 1 e Tabela 1).

**Tabela 1:** Função renal dos diferentes grupos experimentais – São Paulo, SP, Brasil, 2024.

Grupo	N	Creatinina sérica (mg/dL)	n	*Clearance* de inulina (mL/min/100 g)
SHAM	5	0,3 ± 0,1 [0,2–0,4]	5	0,81 ± 0,06 [0,8–0,9]
TCZ	5	0,3 ± 0,1 [0,2–0,4]	5	0,75 ± 0,1 [0,7–0,8]
I/R	5	2,3^a b^ ± 0,6 [1,7–2,8]	5	0,24^a b^ ± 0,03 [0,2–0,3]
TCZ + I/R	5	0,8^c^ ± 0,3 [0,5–1,1]	5	0,65^c^ ± 0,05 [0,6–0,7]

Fonte: Dados da autora, 2024.

Legenda: ^a^p < 0,05 versus SHAM.

^b^versus TCZ.

^c^versus I/R. Valores expressos como média ± desvio padrão (DP), seguidos do intervalo de confiança de 95% (IC95%). Aanálise estatística demonstrou diferenças significativas: creatinina sérica: aumento no grupo I/R (2,3 mg/dL; IC95% [1,7–2,8]) em relação a SHAM e TCZ; redução no grupo TCZ + I/R (0,8 mg/dL; IC95% [0,5–1,1]) em comparação com o grupo I/R. *Clearance* de inulina: redução no grupo I/R (0,24 mL/min/100g; IC95% [0,2–0,3]) em relação a SHAM e TCZ; elevação no grupo TCZ + I/R (0,65 mL/min/100g; IC95% [0,6–0,7]) em comparação com o grupo I/R.

### Perfil Oxidativo

Os parâmetros do perfil oxidativo foram apresentados na Figura 2. Conforme demonstrado, o grupo SHAM foi considerado com valores de normalidade. O grupo TCZ mostrou dados semelhantes aos do grupo SHAM.

**Figura 2:** Estresse oxidativo – São Paulo, SP, Brasil, 2024.

Quanto aos peróxidos urinários, peroxidação lipídica e nitrato urinário, observou-se que o grupo I/R apresentou média elevada desses parâmetros quando comparado aos grupos controle SHAM e TCZ. O grupo TCZ + I/R apresentou redução dos metabólitos oxidativos em comparação com o grupo I/R.

Esse achado demonstra uma resposta oxidativa semelhante entre os grupos controle e o grupo TCZ + I/R. Quanto à resposta antioxidante, os grupos I/R e TCZ + I/R apresentaram valores inferiores (p < 0,05) aos dos grupos controle e praticamente sem diferença entre si (Figura 2 e Tabela 2).

**Tabela 2:** Perfil oxidativo dos animais – São Paulo, SP, Brasil, 2024.

Grupo	n	Peróxidos urinários – FOX 2 (nM/g de creatinina urinária)	n	Peroxidação lipídica – TBARS (nM/g de creatinina urinária)	n	Nitrato urinário (nmol/g creatinina urinária)	n	Tióis (nmol/mg proteína total)
SHAM	5	3,3 ± 0,6	5	0,18 ± 0,01	5	16,0 ± 5,2	5	15,7 ± 6,1
TCZ	5	4,4 ± 0,4	7	0,24 ± 0,13	5	34,9 ± 3,4	5	18,0 ± 4,0
I/R	5	15,8^a b^ ± 2,8	6	1,50^a bd^ ± 0,27	5	174,5^a bd^ ± 13,0	5	1,7^a b^ ± 0,2
TCZ + I/R	5	3,7^c^ ± 1,3	5	0,36^c^ ± 0,26	5	105,3^a b c^ ± 30,8	5	2,5^a b^ ± 0,1

Fonte: São Paulo, SP, Brasil, 2024. Dados da autora.

Legenda: ^a^p < 0,05 versus SHAM

^b^versus TCZ

^c^versus I/R. Dados originais seguidos de média e desvio padrão. A tabela apresenta expressão significativa quanto à análise dos dados dos grupos I/R e TCZ + I/R em: peróxidos urinários (15,8 nM/g de creatinina urinária e 3,7 nM/g de creatinina urinária respectivamente); peroxidação lipídica (1,50 nM/g de creatinina urinária e 0,36 nM/g de creatinina urinária respectivamente); e nitrato urinário (174,5 nM/g de creatinina urinária e 105,3 nM/g de creatinina urinária respectivamente).

## DISCUSSÃO

É importante analisar o recorte deste estudo, no qual foram avaliados parâmetros de função renal (*clearance* de inulina) e marcadores de estresse oxidativo em animais submetidos à injúria renal isquêmica. Os resultados demonstraram que o uso do TCZ, um anticorpo monoclonal humanizado para o receptor da interleucina 6 (IL-6) usado em condições inflamatórias, promoveu atenuação significativa da lesão determinada pela síndrome de isquemia-reperfusão induzida pelo modelo de clampeamento renal, evidenciada pela elevação do *clearance* de inulina e pela redução do estresse oxidativo.

No estudo, os animais foram submetidos a hipoperfusão tecidual, processo denominado isquemia, que é similar ao que ocorre no organismo em processos de sepse, síndrome coronariana aguda e transplante de órgãos, seguida pelo restabelecimento do fluxo sanguíneo denominado reperfusão. Tal procedimento gera um insulto tecidual que desencadeia a ativação de citocinas inflamatórias e estresse oxidativo^(19)^.

Erdem et al. demonstraram que o dano de IRAi é causado por alguns oxidantes, como o monoaldeído, e alguns mediadores inflamatórios, dentre eles NF-κB, TNF-α, IL-6 e IL-1 β. Sabe-se ainda que a IL-6 tem um papel no dano e reparo de células renais e principalmente em alterações imunológicas, metabólicas, isquêmicas ou toxêmicas nos rins^(19)^.

O estudo demonstrou ainda que a ocorrência de IRAi resultou do procedimento de clampeamento dos pedículos renais. Nesse cenário de lesão por hipoperfusão induzida, observouse que, quando instituído o tratamento prévio com TCZ, o comprometimento de função determinado pela isquemia foi atenuado, demonstrado pela elevação do marcador padrãoouro de função renal aplicado nesse estudo, que é o *clearance* de inulina^(20)^.

O papel da IL-6 na função renal e a potencial lacuna de uso farmacológico do tocilizumabe está descrito para os danos renais identificados em glomerulonefrites agudas e em transplantes renais, nos quais ela atua como mecanismo próinflamatório^(21–22)^. A IL-6 desempenha um papel central nesse contexto, uma vez que regula processos inflamatórios, além de mediar a ativação e maturação de células T, células B e plasmócitos – mecanismos intimamente ligados à rejeição. Estudos prévios, como o de Jordan et al. (2017), demonstraram que a inibição dessa citocina reduz significativamente a perda de enxertos, reforçando o potencial terapêutico do TCZ em cenários de injúria renal^(23)^.

Em um modelo experimental realizado na Turquia com ratos submetidos a uma hora de isquemia e seguida por seis horas de reperfusão, dividiram seus animais em três grupos, isquêmicos e isquêmicos tratados com TCZ prévio, observouse que os tratados demonstraram redução nos valores de ureia e creatinina, em consonância com o nosso modelo, que usou o *clearance* de creatinina como marcador de função^(23)^.

Segundo Fukuda (2021), em um relato de caso de pacientes que fizeram uso de tocilizumabe e apresentavam a função renal comprometida, mantiveram suas funções residuais preservadas e permaneceram sem necessidade de terapias de substituição renal, comprovando a proteção renal desse fármaco, contudo, ainda não há consenso sobre o uso profilático do TCZ em doenças renais^(24,25,26,27,28)^. _._Um estudo clínico que utilizou o TCZ reforçou achados promissores em transplantes renais, nos quais a rejeição mediada por anticorpos é uma das principais causas de complicações pós-transplante^(28,29)^.

Nessas condições, os resultados mostrados no presente trabalho corroboram os achados de pesquisas experimentais e clínicas que destacam o tocilizumabe não apenas modulando a inflamação via IL-6, mas também reduzindo significativamente o estresse oxidativo, protegendo contra danos celulares e disfunções vasculares, confirmando que a redução do estresse oxidativo comprovou a redução da inflamação^(28–29)^.

A análise dos marcadores de estresse oxidativo demonstrou valores de normalidade nos grupos SHAM e TCZ e alterações de função renal do grupo I/R. O grupo TCZ + I/R apresentou grande redução no perfil de peroxidação lipídica e peróxidos urinários. Isso pressupõe que o tocilizumabe foi eficaz na redução de EROs e, possivelmente, tenha contribuído para a melhora da resposta mitocondrial em condições de isquemia^(28–29)^. Além disso, à avaliação de nitrato urinário, o grupo TCZ + I/R demonstrou diferença significativa com os grupos controle e com o grupo I/R. Esses achados confirmam a capacidade do tocilizumabe de reduzir espécies reativas de nitrogênio^(28–29)^.

Em contrapartida, alguns autores que estudaram a expressão de *microarray* mostraram que o TCZ, em pacientes tratados com artrite idiopática juvenil, pode diminuir tanto o potencial de membrana quanto a produção de ATP com subsequente aumento no nível de EROS intracelulares. Atualmente, há evidências crescentes de que as EROS mitocondriais e as respostas antioxidantes defeituosas desempenham um papel importante na patogênese de uma série de doenças antinflamatórias e autoimunes^(28,29,30)^.

Dessa forma, o estudo demonstrou que o tocilizumabe se confirma como fármaco com ação antioxidante, com efeito renoprotetor, capaz de atenuar a lesão renal desencadeada pela síndrome isquemia-reperfusão renal, corroborando os achados encontrados na literatura, podendo representar uma estratégia terapêutica em situações em que a isquemia já está instalada, como nos casos de sepse ou mesmo em cirurgias de transplante renal, para atenuar os efeitos lesivos do tempo de isquemia do enxerto e otimizar a recuperação de função no pós-operatório^(29–30)^.

Na esfera da pesquisa experimental, vale ressaltar que a construção desse trabalho gerou evidências na pesquisa básica que servem como um subsídio essencial para a prática de Enfermagem, uma vez que a integração das ciências fundamentais, como fisiologia, biologia molecular e experimentação animal, amplia a capacidade de o enfermeiro interpretar mecanismos fisiopatológicos e de articular intervenções mais seguras e consistentes à beira-leito^(29–30)^.

Portanto, o pertencimento à medicina translacional concerne à prática do enfermeiro e corrobora a fisiologia humana, fruto da pesquisa básica, fortalecendo a tomada de decisão fundamentada em evidências e elevando o padrão de cuidado no manejo da IRA_(29–30)_.

### Limitações

O fato de tratar-se de um estudo com modelo experimental com animais pode restringir sua aplicabilidade imediata na clínica, contudo, os resultados aqui identificados representam subsídio para estudo com humanos, como ensaios randomizados, que possam vir a impactar políticas públicas e resultar na inserção oficial do medicamento e seus similares no arsenal farmacológico nacional, para casos em que a inflamação é o mecanismo determinante de doenças em âmbito nacional.

### Implicações Futuras

As perspectivas futuras desta pesquisa envolvem a expansão dos achados e a elucidação minuciosa dos mecanismos de ação do tocilizumabe na injúria renal aguda isquêmica (IRAi), além disso, a condução de avaliações a longo prazo para investigar o impacto do TCZ na recuperação renal e na prevenção de danos crônicos subsequentes à IRAi em modelos animais e, futuramente, em estudos clínicos.

## CONCLUSÃO

O tocilizumabe mostrou-se promissor na proteção do rim em modelo pré-clínico de isquemia e reperfusão, visto a melhora da função renal demonstrada pela creatinina sérica e pela *clearance* de inulina. Quanto ao perfil oxidativo, esse medicamento mostrou efeito antioxidante, com redução nos peróxidos urinários, na peroxidação lipídica e no nitrato urinário no modelo de IRAi delineado no estudo.

## Data Availability

Todo o conjunto de dados que dá suporte aos resultados deste estudo foi publicado no próprio artigo.

# Translation: Efectos del tocilizumab sobre la función renal y el estrés oxidativo en un modelo experimental de lesión renal aguda isquémica^*^

**DOI:** 10.1590/1980-220X-REEUSP-2025-0220es

**Authors:** Julia Matheus Tsuchiya, Juliana Veloso Gusmão Silva, Mykelly Gomes Alves, Eloiza de Oliveira Silva, Alessandra de Oliveira Maia, Maria de Fátima Fernandes Vattimo

**Affiliations:** 1 Universidade de São Paulo, Escola de Enfermagem, São Paulo, SP, Brasil.

**Keywords:** Lesión Renal Aguda, Anticuerpos Monoclonales, Isquemia

## Abstract

**Introducción:**

La lesión renal aguda (LRA) se deriva de daños renales que desencadenan estrés oxidativo, apoptosis, anomalías estructurales en las células y sus orgánulos, e incluso inestabilidad en el ADN mitocondrial. El tocilizumab es un anticuerpo monoclonal inhibidor de la interleucina-6, utilizado inicialmente para el tratamiento de la artritis reumatoide y, posteriormente, para el tratamiento de la COVID-19 que puede tener un efecto protector en la LRA.

**Objetivo:**

Evaluar el efecto del tocilizumab sobre la función y el perfil oxidativo renal de ratas con LRA isquémica.

**Métodos:**

Se trata de un estudio experimental con animales de enfoque cuantitativo. Los animales fueron aleatorizados en cuatro grupos: SHAM (control); TCZ (tocilizumab); I/R (isquemia/reperfusión renal por pinzamiento de los pedículos renales durante 30 minutos) y TCZ + I/R. Se realizaron pruebas de función renal (aclaramiento de inulina, creatinina plasmática) y oxidación renal (peróxidos urinarios, sustancias oxidativas derivadas del ácido malondialdehído) y agentes antioxidantes endógenos (tioles).

**Resultados:**

En cuanto a la función renal, el grupo tratado presentó un aumento del aclaramiento de inulina **(IR 0,24 ± 0,3 vs TCZ + IR 0,65 ± 0,05; p < 0,05)** y de la creatinina plasmática **(IR 2,3 ± 0,6 vs TCZ + IR 0,8 ± 0,3; p < 0,05)**. El análisis del perfil oxidativo demostró una reducción de los peróxidos, confirmando una atenuación del mecanismo redox **(IR 15,8 ± 2,8 vs TCZ + IR 3,7 ± 1,3; p < 0,05)**.

**Conclusión:**

El tocilizumab presentó un efecto renoprotector, con una mejora de la función renal y una reducción del estrés oxidativo.

## INTRODUCCIÓN

La lesión renal aguda (LRA) es la reducción abrupta de la función renal, definida por la disminución de la tasa de filtración glomerular (TFG), lo que da lugar a un aumento transitorio de los desechos renales, como la urea y la creatinina, y puede ir acompañada de una reducción del flujo urinario y de desequilibrios hidroelectrolíticos^(1)^.

Entre sus clasificaciones, según la etiología, se describen la prerrenal y la renal, que pueden estar asociadas a eventos de isquemia, como sepsis, choque, infecciones, uso de contrastes radiológicos y toxicidad por medicamentos^(2)^. Según la organización Kidney Disease: improving global outcomes (KDIGO), 1 de cada 5 adultos (21,6%) y 1 de cada 3 niños (33,7%) desarrollan la LRA en el mundo. Clínicamente, este síndrome se define como un aumento de 0,3 mg/dl o 1,5 veces en la creatinina sérica basal en 48 horas o una reducción del flujo urinario a menos de 0,5 ml/kg durante 6 horas^(3)^.

En situaciones de isquemia en las que se producen períodos prolongados de reducción del flujo sanguíneo que causan hipoperfusión renal, como en casos de sepsis, cirugías cardiovasculares o trasplantes de aloinjertos, se produce un compromiso de la integridad y el metabolismo celular renal y estrés oxidativo y, en última instancia, disfunciones vasculares, glomerulares y tubulares, lo que genera un daño en este órgano^(3,4)^.

La lesión por isquemia es la causa de la reducción de la TFG, pero esta no siempre se relaciona directamente con la condición basal del paciente, lo que demuestra la complejidad de los procesos vasculares y tubulares en la disfunción renal^(4)^.

Algunos estudios proponen un procedimiento de mimetización de la Lesión Renal Aguda generada por Isquemia (la LRAi) que consiste en la hipoxia de los tejidos renales, causada por la interrupción de la irrigación sanguínea local. En este caso, la isquemia se induce mediante el pinzamiento de los pedículos renales, seguido de la reperfusión del flujo sanguíneo en el tejido renal^(4)^.

Esta técnica de lesión renal causa daños mitocondriales, ya que estas organelas dependen del oxígeno para llevar a cabo los procesos de fosforilación oxidativa, el principal mecanismo de producción de energía. En ausencia o supresión del oxígeno, este proceso se produce de forma anaeróbica y da lugar a la formación de productos como el ácido láctico. Este ácido, cuando se acumula, genera acidosis celular y puede causar daños en la estructura de la membrana mitocondrial^(5–6)^. Este conjunto de daños celulares da lugar a inflamación, consecuencia de la activación del sistema inmunitario proinflamatorio cercano al lugar^(7)^.

En lo que respecta a las células de defensa y los agentes inflamatorios, destacan los leucocitos, los mastocitos y las plaquetas, que liberan varios tipos de mediadores lipídicos (eicosanoides), proteínas (citocinas y quimiocinas) y mediadores gaseosos (óxido nítrico, monóxido de carbono, especies reactivas de oxígeno)^(7)^. La inflamación se ha considerado el principal mecanismo de lesión en lesiones isquémicas como la LRAi y está directamente relacionada con el mecanismo redox.

En la LRAi, la interleucina-6 (IL-6) desempeña un papel importante tanto en la fase inicial de la inflamación como en la fase de recuperación. Durante la isquemia, se produce una respuesta inflamatoria significativa en la que la IL-6 es producida rápidamente por las células endoteliales, los macrófagos y las células epiteliales renales. La producción de IL-6 es estimulada por señales locales de estrés celular e hipoxia, lo que promueve la activación de leucocitos y la liberación de otras citocinas proinflamatorias^(7–8)^.

El mecanismo inflamatorio en la LRAi implica un aumento de la infiltración de neutrófilos y macrófagos en los riñones lesionados, lo que agrava el daño tisular. La vía de transseñalización de la IL-6, mediada por el receptor soluble de IL-6 (sIL-6R), está particularmente asociada con la intensificación del proceso inflamatorio, agravando la lesión renal al amplificar la respuesta inmunitaria y causar daño oxidativo y apoptosis en las células renales^(8–9)^.

En este sentido, la hipótesis de este estudio es que el tocilizumab (TCZ) puede prevenir la LRA por su acción inhibidora sobre la IL-6, interfiriendo tanto en las respuestas inmunológicas específicas a los antígenos como en los procesos inflamatorios, posicionándose como uno de los principales agentes de la etapa inicial de la inflamación^(8)^.

En resumen, el TCZ es un anticuerpo monoclonal inhibidor de la IL-6. Inicialmente se utilizó para el tratamiento de la artritis reumatoide y, posteriormente, se probó para el tratamiento de la infección respiratoria aguda causada por el coronavirus SARS-CoV-2 (COVID-19), por sus efectos en el perfil de marcadores inflamatorios, como la proteína C reactiva, la ferritina y la deshidrogenasa láctica en pacientes con cánula nasal de alto flujo y ventilación no invasiva^(8–9)^.

El estudio que aquí se presenta sigue un modelo de investigación preclínica cuyos resultados pueden respaldar las decisiones asistenciales tras su incorporación en estudios clínicos y ensayos aleatorios, lo que puede dar lugar a mejoras relevantes en la práctica clínica^(10,11,12)^.

El enfermero investigador desempeña un papel fundamental en la medicina traslacional, al desarrollar y probar modelos experimentales e innovaciones cuyo objetivo final es la aplicación práctica y la mejora de los resultados clínicos, como en el caso de los nuevos fármacos, para preservar la función renal, al igual que en el proyecto que se presenta^(13)^.

El dominio de las ciencias básicas, por lo tanto, es esencial tanto para la formación de nuevos investigadores como para la práctica avanzada, ampliando el modelo tradicional de «cuidado» y legitimando la participación de la enfermería en la creación de terapias innovadoras^(13)^. Por lo tanto, la presente investigación tuvo como objetivo evaluar el efecto del tocilizumab sobre la función y el perfil oxidativo renal de ratas con la LRA isquémica.

## MÉTODO

### Aspectos Éticos

El estudio fue aprobado por el Comité de Ética para el Uso de Animales de la Facultad de Medicina de la Universidad de São Paulo (CEUA-FMUSP), con el registro CEUA: 2013/2023. Se utilizaron 20 ratas Wistar, machos y adultos, proporcionadas por la Facultad de Medicina de la USP. El estudio se llevó a cabo en el Laboratorio Experimental de Modelos Animales (LEMA) de la Escuela de Enfermería de la Universidad de São Paulo (EEUSP). El aspecto ético del protocolo experimental se basó en la Directriz Brasileña para el Cuidado y la Utilización de Animales con Fines Científicos y Didácticos (DBCA). De conformidad con los preceptos de la Ley n.º 11.794, de 8 de octubre de 2008, el Decreto n.º 6.899, de 15 de julio de 2009, y las normas editadas por el Consejo Nacional de Control de Experimentación en Animales (CONCEA) y la guía ARRIVE 2.0^(14)^.

### Diseño Experimental

El cálculo de la muestra utilizado en el estudio se realizó mediante el software G*power*, que* recomienda el número de animales necesarios para el experimento. Las ratas Wistar, machos y adultos, pesaban entre 250 y 300 g. Los animales se distribuyeron aleatoriamente en cuatro grupos: Grupo SHAM (n = 5): animales control sometidos a laparotomía para simular el pinzamiento de los pedículos renales; Tocilizumab (TCZ, n = 5): animales que recibieron tocilizumab (4 mg/kg, i.p., dosis única) el primer día del protocolo experimental; Isquemia y reperfusión (I/R, n = 5): animales sometidos a laparotomía para el pinzamiento bilateral de los pedículos renales durante 30 minutos; y TCZ + I/R (n = 5): animales que recibieron tocilizumab (4 mg/kg, i.p., dosis única) el primer día del protocolo y, el segundo día, fueron sometidos al procedimiento de I/R.

Los animales recibieron un preanestésico con morfina (2%, 2 mg/kg, una vez, intramuscular) y, tras 10 minutos, fueron anestesiados con isoflurano (5% dosis de inducción y 3% dosis de mantenimiento a 2 L de O_2_) y sometidos a laparotomía para el pinzamiento bilateral de los pedículos renales con *pinzas* vasculares no traumáticas. Todos los animales fueron suturados con hilo *mononylon* 3.0, acompañados durante la recuperación anestésica y recibieron un protocolo de analgesia postoperatoria con tramadol (15 mg/kg intramuscular, 3 veces al día, 3 días). Al tercer día, fueron colocados en una jaula metabólica para analizar la función renal y el perfil oxidativo.

El cuarto día, los animales fueron anestesiados como se ha descrito anteriormente (morfina seguida de isoflurano) y colocados en una mesa quirúrgica calefactada. Se realizó una traqueotomía para liberar las vías aéreas superiores. A continuación, se procedió a la disección de la vena yugular izquierda para la infusión de inulina durante 2 horas y la recolección de muestras de sangre cada 60 minutos, además de la canulación de la carótida directa para medir la presión arterial media. También se realizó una laparotomía para drenar la vejiga y recoger muestras de orina cada 30 minutos, seguida de la disección o punción de la aorta abdominal. Se extrajo el riñón izquierdo, se acondicionó y se almacenó en un refrigerador a -80 °C para estudios posteriores de medición de tioles no proteicos.

### Evaluación del Estrés Oxidativo

Las evaluaciones se realizaron en muestras de plasma, orina y tejido renal extraídas y almacenadas después de la eutanasia, siguiendo los siguientes análisis:

Peróxidos urinarios: se evaluaron mediante el ensayo de oxidación ferrosa de naranja de xilenol (FOX), que mide directamente los peróxidos y consiste en el uso de hierro-xilenol naranja para determinar los niveles de peróxidos urinarios^(15–16)^.

Peroxidación lipídica: se analizó mediante el ensayo de sustancias reactivas al ácido tiobarbitúrico (TBARS), que mide el malondaldehído (MDA) por ser uno de los principales productos de esta cascada. Un método para detectar este producto es la dosificación del ácido tiobarbitúrico al que reacciona^(17–18)^.

Síntesis de nitrato urinario: se evaluó mediante la cuantificación de nitrito (NO_2_^-^), metabolito estable del NO, por el método de Griess. La reacción es colorimétrica y se basa en la reacción de los nitritos con el ácido sulfanílico y la copulación con clorhidrato de alfa-naftilamina en medio ácido (pH entre 2,5 y 5,0), formando el ácido alfa-naftilamino-p-azobenceno-p-sulfónico de coloración rosada. Se añadieron aproximadamente 150 µl de la muestra de orina de los distintos grupos a 150 µl del reactivo, que se dejó reposar durante 15 minutos. La lectura se realizó en absorbancia a 545 nm, en un lector ELISA. La absorbancia de las muestras se comparó con una curva estándar de nitrito de sodio 15 (NaNO^2^) en una concentración de 0,1 a 1,0 M^(18)^.

Cuantificación de tioles (antioxidantes): se utilizó como indicador de respuesta antioxidante, considerando el siguiente principio: cuanto mayor era el grado de estrés oxidativo, mayores eran los niveles de tioles oxidados y menores eran las concentraciones de tioles en el tejido renal^(18)^.

### Análisis Estadístico

El análisis de los datos se realizó con el software GraphPad Prism versión 8.0, mediante el método ANOVA. Se adoptó un nivel de significación de p < 0,05, considerando que el efecto de al menos uno de los grupos difería de los demás. Y las pruebas múltiples de comparación 2 a 2 realizadas mediante la prueba de Tukey, comparando los posibles pares de medias basándose en la diferencia mínima significativa en la homogeneidad de las varianzas que difieren o no entre sí en los grupos.

## RESULTADOS

### Parámetros de Función Renal

Los parámetros de función renal se presentan en la Figura 1. Los datos del SHAM se utilizaron como grupo control, considerados referencia de normalidad. Siguiendo esta misma lógica, el grupo TCZ presentó datos de función renal (clearance de inulina) similares a los demostrados por el grupo SHAM, lo que confirma que el tocilizumab, por sí solo, no afectó a la función renal.

El grupo I/R confirmó la LRA considerando el aumento de la creatinina y la disminución del clearance de inulina (p < 0,05) (Tabla 1 y Figura 1). El grupo TCZ + I/R mostró una reducción de la creatinina plasmática (p < 0,05) y un aumento significativo del clearance de inulina en comparación con el grupo I/R (Figura 1 y Tabla 1).

**Figura 1:** Función renal de los diferentes grupos experimentales – São Paulo, SP, Brasil, 2024.

**Tabla 1.** Función renal de los diferentes grupos experimentales – São Paulo, SP, Brasil, 2024

Grupo	N	Creatinina sérica (mg/dL)	n	Clearance de inulina (mL/min/100 g)
SHAM	5	0,3 ± 0,1 [0,2–0,4]	5	0,81 ± 0,06 [0,8–0,9]
TCZ	5	0,3 ± 0,1 [0,2–0,4]	5	0,75 ± 0,1 [0,7–0,8]
I/R	5	2,3^a b^ ± 0,6 [1,7–2,8]	5	0,24^a b^ ± 0,03 [0,2–0,3]
TCZ + I/R	5	0,8^c^ ± 0,3 [0,5–1,1]	5	0,65^c^ ± 0,05 [0,6–0,7]

Fuente: datos de la autora, 2024.

Claves: ^a^p < 0,05 frente a SHAM.

^b^frente a TCZ.

^c^frente a I/R. Valores expresados como media ± desviación estándar (DE), seguidos del intervalo de confianza del 95% (IC95%). El análisis estadístico demostró diferencias significativas: creatinina sérica: aumento en el grupo I/R (2,3 mg/dL; IC95% [1,7–2,8]) en relación con SHAM y TCZ; reducción en el grupo TCZ + I/R (0,8 mg/dL; IC95% [0,5–1,1]) en comparación con el grupo I/R. *Clearance* de inulina: reducción en el grupo I/R (0,24 mL/min/100g; IC95% [0,2–0,3]) en relación con SHAM y TCZ; aumento en el grupo TCZ + I/R (0,65 mL/min/100 g; IC95% [0,6–0,7]) en comparación con el grupo I/R.

### Perfil Oxidativo

Los parámetros del perfil oxidativo se presentan en la Figura 2.

**Figura 2:** Estrés oxidativo – São Paulo, SP, Brasil, 2024.

Como se muestra, el grupo SHAM se consideró con valores normales. El grupo TCZ mostró datos similares a los del grupo SHAM. En cuanto a los peróxidos urinarios, la peroxidación lipídica y el nitrato urinario, se observó que el grupo I/R presentó un promedio elevado de estos parámetros en comparación con los grupos control SHAM y TCZ.

El grupo TCZ + I/R presentó una reducción de los metabolitos oxidativos en comparación con el grupo I/R.

Este hallazgo demuestra una respuesta oxidativa similar entre los grupos control y el grupo TCZ + I/R. En cuanto a la respuesta antioxidante, los grupos I/R y TCZ + I/R presentaron valores inferiores (p < 0,05) a los de los grupos control y prácticamente sin diferencia entre sí (Figura 2 y Tabla 2).

**Tabla 2:** Perfil oxidativo de los animales – São Paulo, SP, Brasil, 2024.

Grupo	n	Peróxidos urinarios – FOX 2 (nM/g de creatinina urinaria)	N	Peroxidación lipídica – TBARS (nM/g de creatinina urinaria)	n	Nitrato urinario (nmol/g creatinina urinaria)	n	Tioles (nmol/mg proteína total)
SHAM	5	3,3 ± 0,6	5	0,18 ± 0,01	5	16,0 ± 5,2	5	15,7 ± 6,1
TCZ	5	4,4 ± 0,4	7	0,24 ± 0,13	5	34,9 ± 3,4	5	18,0 ± 4,0
I/R	5	15,8^a b^ ± 2,8	6	1,50^a bd^ ± 0,27	5	174,5^a bd^ ± 13,0	5	1,7^a b^ ± 0,2
TCZ + I/R	5	3,7^c^ ± 1,3	5	0,36^c^ ± 0,26	5	105,3^a b c^ ± 30,8	5	2,5^a b^ ± 0,1

Fuente: São Paulo, SP, Brasil, 2024.

Datos de la autora. Claves: ^a^p < 0,05

frente a SHAM; ^b^frente a TCZ;

^c^frente a I/R. Datos originales seguidos de la media y la desviación estándar. La tabla presenta una expresión significativa en cuanto al análisis de los datos de los grupos I/R y TCZ + I/R en: peróxidos urinarios (15,8 nM/g de creatinina urinaria y 3,7 nM/g de creatinina urinaria, respectivamente); peroxidación lipídica (1,50 nM/g de creatinina urinaria y 0,36 nM/g de creatinina urinaria, respectivamente); y nitrato urinario (174,5 nM/g de creatinina urinaria y 105,3 nM/g de creatinina urinaria, respectivamente).

## DISCUSIÓN

Es importante analizar el enfoque de este estudio, en el que se evaluaron los parámetros de la función renal (clearance de inulina) y los marcadores de estrés oxidativo en animales sometidos a lesión renal isquémica.

Los resultados demostraron que el uso de TCZ, un anticuerpo monoclonal humanizado para el receptor de interleucina 6 (IL-6) utilizado en condiciones inflamatorias, promovió una atenuación significativa de la lesión determinada por el síndrome de isquemia-reperfusión inducido por el modelo de pinzamiento renal, evidenciada por el aumento del clearance de inulina y la reducción del estrés oxidativo.

En el estudio, los animales fueron sometidos a hipoperfusión tisular, un proceso denominado isquemia, similar al que se produce en el organismo en procesos de sepsis, síndrome coronario agudo y trasplante de órganos, seguido del restablecimiento del flujo sanguíneo denominado reperfusión. Este procedimiento genera una agresión tisular que desencadena la activación de citocinas inflamatorias y estrés oxidativo^(19)^.

Erdem et al. demostraron que el daño de la LRAi es causado por algunos oxidantes, como el mono aldehído, y algunos mediadores inflamatorios, entre ellos NF-κB, TNF-α, IL-6 e IL-1 β. También se sabe que la IL-6 desempeña un papel en el daño y la reparación de las células renales y, principalmente, en las alteraciones inmunológicas, metabólicas, isquémicas o toxémicas en los riñones^(19)^.

El estudio también demostró que la aparición de la LRA fue resultado del procedimiento de pinzamiento de los pedículos renales. En este escenario de lesión por hipoperfusión inducida, se observó que, cuando se instauró el tratamiento previo con TCZ, el compromiso de la función determinado por la isquemia se atenuó, lo que se demostró por la elevación del marcador de referencia de la función renal aplicado en este estudio, que es el *clearance de* inulina^(20)^.

El papel de la IL-6 en la función renal y la posible laguna en el uso farmacológico del tocilizumab se describe para los daños renales identificados en glomerulonefritis agudas y en trasplantes renales, en los que actúa como mecanismo proinflamatorio^(21–22)^. La IL-6 desempeña un papel central en este contexto, ya que regula los procesos inflamatorios, además de mediar la activación y maduración de las células T, las células B y los plasmocitos, mecanismos íntimamente relacionados con el rechazo. Estudios previos, como el de Jordan et al. (2017), han demostrado que la inhibición de esta citocina reduce significativamente la pérdida de injertos, lo que refuerza el potencial terapéutico del TCZ en escenarios de lesión renal^(23)^.

En un modelo experimental realizado en Turquía con ratas sometidas a una hora de isquemia seguida de seis horas de reperfusión, se dividieron los animales en tres grupos, isquémicos e isquémicos tratados previamente con TCZ, y se observó que los tratados mostraron una reducción en los valores de urea y creatinina, en consonancia con nuestro modelo, que utilizó el clearance de creatinina como marcador de la función^(23)^.

Según Fukuda (2021), en un informe de casos de pacientes que utilizaron tocilizumab y presentaban una función renal comprometida, mantuvieron sus funciones residuales preservadas y no necesitaron terapias de sustitución renal, lo que demuestra la protección renal de este fármaco. Sin embargo, aún no hay consenso sobre el uso profiláctico del TCZ en enfermedades renales^(24,25,26,27,28)^.Un estudio clínico que utilizó TCZ reforzó los prometedores hallazgos en trasplantes renales, en los que el rechazo mediado por anticuerpos es una de las principales causas de complicaciones postrasplante^(28–29)^.

En estas condiciones, los resultados mostrados en el presente trabajo corroboran los hallazgos de investigaciones experimentales y clínicas que destacan que el tocilizumab no solo modula la inflamación a través de la IL-6, sino que también reduce significativamente el estrés oxidativo, protegiendo contra el daño celular y las disfunciones vasculares, lo que confirma que la reducción del estrés oxidativo demostró la reducción de la inflamación^(28–29)^.

El análisis de los marcadores de estrés oxidativo mostró valores normales en los grupos SHAM y TCZ y alteraciones de la función renal en el grupo I/R. El grupo TCZ + I/R presentó una gran reducción en el perfil de peroxidación lipídica y peróxidos urinarios. Esto supone que el tocilizumab fue eficaz en la reducción de las ERO y, posiblemente, contribuyó a la mejora de la respuesta mitocondrial en condiciones de isquemia^(28–29)^. Además, en la evaluación del nitrato urinario, el grupo TCZ+I/R mostró una diferencia significativa con los grupos control y con el grupo I/R. Estos hallazgos confirman la capacidad del tocilizumab para reducir las especies reactivas de nitrógeno^(28–29)^.

Por el contrario, algunos autores que estudiaron la expresión del *microarray* demostraron que el TCZ, en pacientes tratados con artritis idiopática juvenil, puede disminuir tanto el potencial de membrana como la producción de ATP, con el consiguiente aumento del nivel de ROS intracelulares. Actualmente, hay cada vez más pruebas de que el estrés oxidativo mitocondrial y las respuestas antioxidantes defectuosas desempeñan un papel importante en la patogénesis de una serie de enfermedades antiinflamatorias y autoinmunes^(28–29,30)^.

De este modo, el estudio demostró que el tocilizumab se confirma como un fármaco con acción antioxidante y efecto renoprotector, capaz de atenuar la lesión renal desencadenada por el síndrome de isquemia-reperfusión renal, lo que corrobora los hallazgos encontrados en la literatura y puede representar una estrategia terapéutica en situaciones en las que la isquemia ya está instalada, como en los casos de sepsis o incluso en cirugías de trasplante renal, para atenuar los efectos lesivos del tiempo de isquemia del injerto y optimizar la recuperación de la función en el posoperatorio^(29–30)^.

En el ámbito de la investigación experimental, cabe destacar que la elaboración de este trabajo generó evidencias en la investigación básica que sirven como un subsidio esencial para la práctica de la enfermería, ya que la integración de las ciencias fundamentales, como la fisiología, la biología molecular y la experimentación animal, amplía la capacidad del enfermero para interpretar los mecanismos fisiopatológicos y articular intervenciones más seguras y consistentes a pie de cama^(29–30)^.

Por lo tanto, la pertenencia a la medicina traslacional concierne a la práctica de la enfermería y corrobora la fisiología humana, fruto de la investigación básica, para fortalecer la toma de decisiones basada en la evidencia y elevar el nivel de atención en el manejo de la LRA^(29–30)^.

### Limitaciones

El hecho de que se trate de un estudio con un modelo experimental con animales puede restringir su aplicabilidad inmediata en la clínica; sin embargo, los resultados aquí identificados representan un aporte para estudios con humanos, como ensayos aleatorios, que pueden llegar a impactar en las políticas públicas y dar lugar a la inclusión oficial del medicamento y sus similares en el arsenal farmacológico nacional, para casos en los que la inflamación es el mecanismo determinante de enfermedades a nivel nacional.

### Implicaciones Futuras

Las perspectivas futuras de esta investigación implican la ampliación de los hallazgos y la elucidación minuciosa de los mecanismos de acción del tocilizumab en la lesión renal isquémica aguda (LRAi), además de la realización de evaluaciones a largo plazo para investigar el impacto del TCZ en la recuperación renal y la prevención de daños crónicos posteriores a la LRAi en modelos animales y, en el futuro, en estudios clínicos.

## CONCLUSIÓN

El tocilizumab se ha mostrado prometedor en la protección renal en un modelo preclínico de isquemia y reperfusión, dada la mejora de la función renal demostrada por la creatinina sérica y el *clearance* de inulina. En cuanto al perfil oxidativo, este medicamento mostró un efecto antioxidante, con una reducción de los peróxidos urinarios, la peroxidación lipídica y el nitrato urinario en el modelo de la LRAi delineado en el estudio.

## Data Availability

Todo el conjunto de datos que apoya los resultados de este estudio fue publicado en el propio artículo.
